# Optogenetic Low-Frequency Stimulation of Principal Neurons, but Not Parvalbumin-Positive Interneurons, Prevents Generation of Ictal Discharges in Rodent Entorhinal Cortex in an In Vitro 4-Aminopyridine Model

**DOI:** 10.3390/ijms24010195

**Published:** 2022-12-22

**Authors:** Elena Y. Proskurina, Anton V. Chizhov, Aleksey V. Zaitsev

**Affiliations:** 1 Almazov National Medical Research Centre, 2 Akkuratova Street, 197341 St. Petersburg, Russia; 2 Sechenov Institute of Evolutionary Physiology and Biochemistry of the Russian Academy of Sciences, 44 Toreza Prospekt, 194223 St. Petersburg, Russia; 3 Computational Physics Laboratory, Ioffe Institute, 26 Polytekhnicheskaya Street, 194021 St. Petersburg, Russia; 4 MathNeuro Team, Inria Centre at Universite Cote d’Azur, 06902 Sophia Antipolis, France

**Keywords:** temporal lobe epilepsy, 4-aminopyridine model, ictal discharge, channelrhodopsin, Thy1-ChR2-YFP line 18 mouse

## Abstract

Low-frequency electrical stimulation is used to treat some drug-resistant forms of epilepsy. Despite the effectiveness of the method in suppressing seizures, there is a considerable risk of side effects. An optogenetic approach allows the targeting of specific populations of neurons, which can increase the effectiveness and safety of low-frequency stimulation. In our study, we tested the efficacy of the suppression of ictal activity in entorhinal cortex slices in a 4-aminopyridine model with three variants of low-frequency light stimulation (LFLS): (1) activation of excitatory and inhibitory neurons (on Thy1-ChR2-YFP mice), (2) activation of inhibitory interneurons only (on PV-Cre mice after virus injection with channelrhodopsin2 gene), and (3) hyperpolarization of excitatory neurons (on Wistar rats after virus injection with archaerhodopsin gene). Only in the first variant did simultaneous LFLS of excitatory and inhibitory neurons replace ictal activity with interictal activity. We suggest that LFLS caused changes in the concentration gradients of K^+^ and Na^+^ cations across the neuron membrane, which activated Na-K pumping. According to the mathematical modeling, the increase in Na-K pump activity in neurons induced by LFLS led to an antiepileptic effect. Thus, a less specific and generalized optogenetic effect on entorhinal cortex neurons was more effective in suppressing ictal activity in the 4-aminopyridine model.

## 1. Introduction

Deep-brain stimulation is an effective method for treating some neurological disorders, including Parkinson’s disease [1], obsessive-compulsive disorder [2], dystonia [3], and Tourette syndrome [4]. Multiple studies in animal models of epilepsy have shown the effectiveness of low-frequency electric stimulation in preventing ictal activity, both in in vivo models [5,6,7] and in vitro models [8,9]. For instance, in a 4-aminopyridine (4-AP) model, the epileptiform activity in brain slices could be prevented by 1 Hz of electrical stimulation; however, every electric stimulus led to an interictal event [8]. Rashid et al. reported that, in a rat model of chronic temporal lobe epilepsy, a 2-week 1 Hz electric stimulation of the ventral hippocampal commissure reduced seizure frequencies by 90% and interictal spike frequency by 71% [5]. In 2018, deep-brain stimulation was approved by the Food and Drug Administration as a treatment for pharmacoresistant epilepsy [10].

Several clinical studies have shown the efficacy of deep-brain electric stimulation [11,12], but there are essential side effects in some cases, including implant site pain and infection [13], paresthesia [14], and memory impairment [15]. Therefore, the study of the antiepileptic mechanism of deep-brain stimulation and the search for the most effective and safe methods of stimulation to suppress ictal activity remain urgent tasks.

To reduce the risk of side effects, more targeted stimulation should be used. This possibility has emerged as a result of the development of the optogenetic approach [16]. The optogenetic approach makes it possible to control a particular type of neuron by the photostimulation of a certain wavelength if a specific rhodopsin is expressed in neurons of this type. The effect of low-frequency light stimulation (LFLS) on epileptiform activity has already been studied in several in vitro and in vivo models. Ladas et al. showed that 1 Hz of photostimulation of Thy1-ChR2-, as well as VGAT-ChR2-, positive neurons suppressed interictal activity in the CA3 zone of the hippocampus induced by the local application of 4-aminopiridine (4-AP) in vivo [17]. Using an immunohistochemical analysis, the authors showed that, in the CA3 region of the hippocampus of Thy1-ChR2- and VGAT-ChR2-YFP mice, the yellow fluorescent protein was often colocalized with GAD-67. Thus, light caused the excitation of interneurons in the CA3 area of the hippocampus in both lines of mice, which prevented the occurrence of interictal discharge [17].

The effect of short-time LFLS on ictal activity was considered in a 4-AP model in slices of the entorhinal cortex and hippocampus of CamkII-Cre, PV-Cre, and SOM-Cre juvenile mice of 2–3 weeks of age after an injection of the virus construct AAV-ChETA-eYFP [18]. Monitoring of the ictal activity was implemented with extracellular recordings. The 1 Hz photostimulation during 180 s of either CaMKII-, SOM-, or PV-positive neurons suppressed ictal activity. The effects of longer durations of LFLS were not reported in that work.

The published studies indicate that the optogenetic approach may be promising in the treatment of pharmacoresistant forms of epilepsy. However, questions remain as to whether the local stimulation of entorhinal cortex neurons effectively suppresses ictal activity. Is the LFLS of adult rodent entorhinal cortex neurons as effective in preventing ictal discharges as low-frequency electrical stimulation? What is the mechanism of possible antiepileptic action? Will the LFLS of certain types of neurons be effective, and what is the effect of the low-frequency hyperpolarization of principal neurons on epileptic activity?

**Figure 1:**
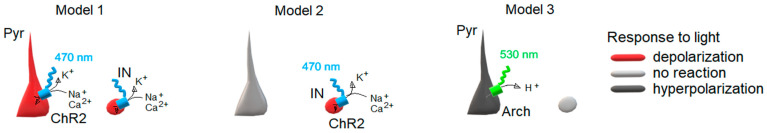
Genetic models used to search for a targeted antiepileptic stimulation. Model 1: activation of both pyramidal cells (Pyrs) and interneurons (INs) expressing Channelrhodopsin2 (ChR2) with 470 nm of photostimulation. ChR2 is a nonspecific cation channel that opens when exposed to light. This results in depolarization and the generation of action potentials in the neurons. Model 2: specific activation of interneurons expressing ChR2 with photostimulation. Model 3: specific hyperpolarization of pyramidal cells expressing Archaerhodopsin (Arch). Arch acts as a transporter, pumping protons out of neurons.

In this work, we try to answer these questions and study the antiepileptic effect of LFLS using three optogenetic models (Figure 1).

Model 1: Thy1-ChR2-YFP line 18 mice. Both pyramidal cells and fast-spiking interneurons in deep layers of the entorhinal cortices of these mice depolarized and fired in response to 470 nm of photostimulation [19].

Model 2: PV-Cre mice line (JAX stock #017320, B6.129P2-Pvalb^tm1(cre)Arbr^/J) after injection with the virus construct AAV9-EF1α-DIO-hChR2(H134R)-mCherry (from Addgene plasmid #20297) to activate PV-expressing interneurons with 470 nm of photostimulation. A similar model was used in another study [18].

Model 3: Wistar rats after injection with the virus construct AAV9-CamkIIa-eArch 3.0-EYFP (from Addgene plasmid #35516) to hyperpolarize CaMKII neurons with 530 nm of photostimulation.

## 2. Results

### 2.1. Epileptiform Activity Was Reliably Induced by 4-AP in Slices of Entorhinal Cortex and Hippocampus of Used Animal Models

**Figure A1:**
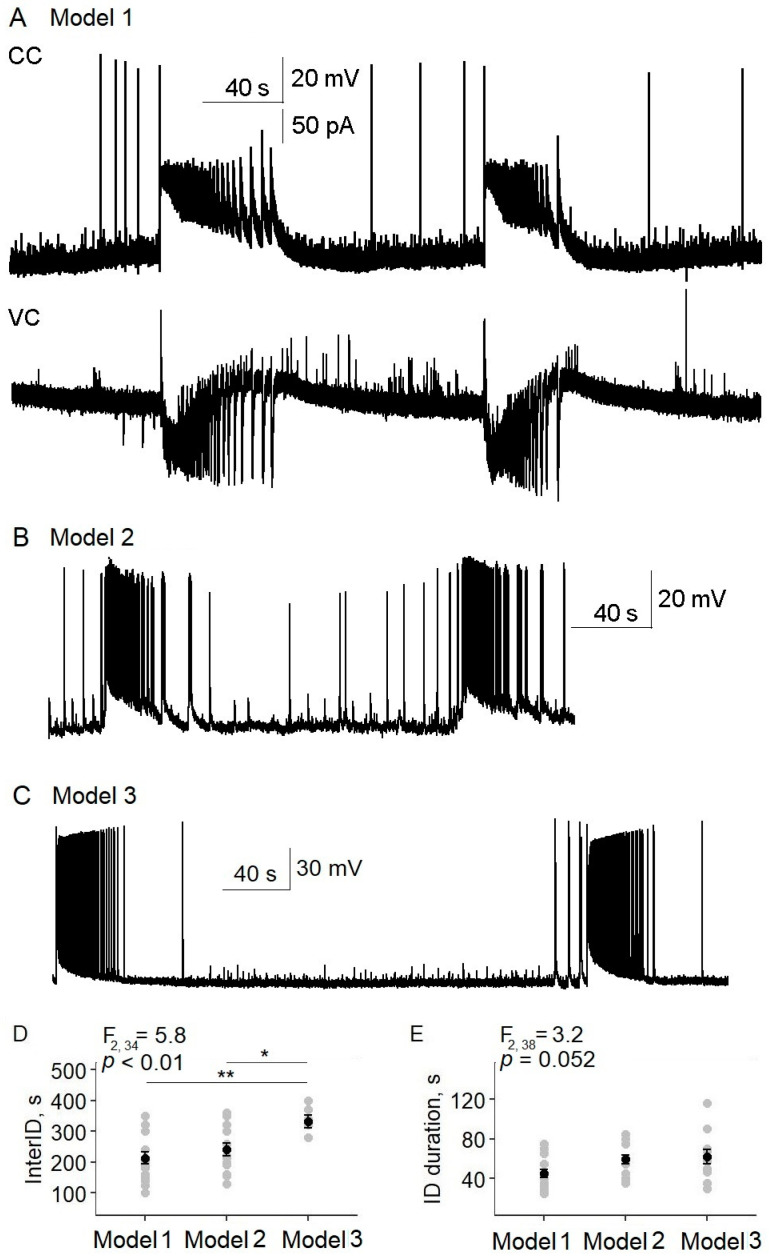
Stable ictal activity was observed in the entorhinal cortices of all three models: (**A**) Thy1-ChR2-YFP and (**B**) PV-Cre mice and (**C**) Wistar rats in 4-AP solution. (**D**) The intervals between ictal discharges (InterID) were the same for mice but less than that for rats (one-way ANOVA with post hoc Tukey test). (**E**) The duration of the ictal discharges did not differ. The InterIDs were 213 ± 19 (*n* = 17), 241 ± 20 (*n* = 14), and 332 ± 20 s (*n* = 6) for Models 1–3, respectively; ID durations were 45 ± 4 (*n* = 15), 59 ± 5 (*n* = 15), and 62 ± 7 s (*n* = 11) for Models 1–3, respectively. * *p* < 0.05, ** *p* < 0.01

Initially, we confirmed that stable ictal activity was induced in the entorhinal cortices of all three models with the epileptogenic 4-AP solution (Appendix A). The ictal discharge had a classic tonic–clonic structure beginning from inhibitory postsynaptic currents (IPSCs), but then the excitatory postsynaptic currents (EPSCs) prevailed (Figure A1). Sometimes, short epileptiform events called interictal discharges were also recorded. The epileptiform activity is similar to those observed in our previous works [20,21] and in studies of other authors [9,22].

### 2.2. LFLS of Excitatory and Inhibitory Neurons Prevented Generation of Ictal Discharges

**Figure 2:**
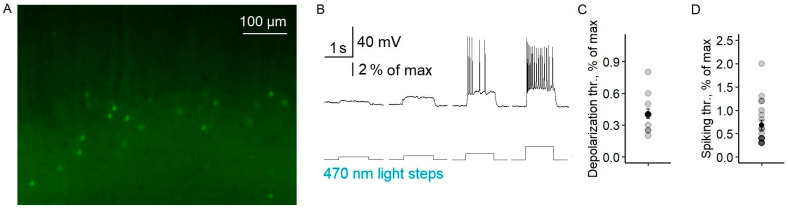
The electrophysiological properties of ChR2-expressing neurons (Model 1). (**A**) Microphotography of the deep layers of the entorhinal cortex. (**B**) A representative example of the responses of a pyramidal neuron to light steps with different intensities. Depolarization (**C**) and spiking (**D**) threshold LI in pyramidal cells. Circles are individual values, small black circles are mean values, and whiskers are standard errors of the means. Light intensity was calculated in percentages of the maximal value of LED.

Using Model 1, we investigated the antiepileptic effects of the LFLS of both excitatory and inhibitory neurons in a 4-AP solution. First, we confirmed that both types of neurons responded to light (Figure 2A; see also [19]). The threshold light intensity (LI) causing the depolarization of pyramidal neurons was 0.41 ± 0.04% of the maximum intensity (*n* = 13, Figure 2B,C). The firing activity of pyramidal neurons was induced by 0.68 ± 0.10% of the maximum LI (*n* = 20, Figure 2B,D). The fast-spiking interneurons started firing at a lower LI than the pyramidal cells (0.39 ± 0.03%, *n* = 10, *t*-test, *p* < 0.05), as we previously showed [19]. An increase in light intensity raised the frequency of neuron action potentials (Appendix B). Thus, short flashes of light reliably induced spikes in both interneurons and pyramidal neurons.

**Figure 3:**
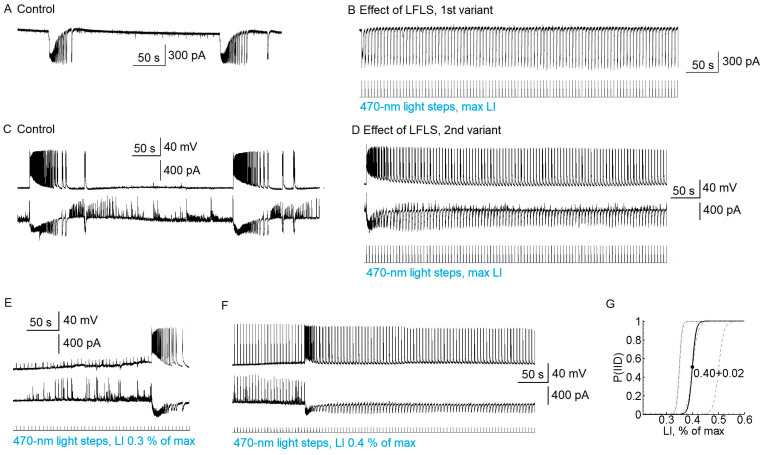
LFLS of excitatory and inhibitory neurons in the entorhinal cortex (Model 1) prevented the generation of ictal discharges but induced interictal discharges; only the first light step could induce ictal discharge. (**A**,**B**) Representative recordings of the synaptic currents of a pyramidal cell in the control group (**A**) and under LFLS with maximal intensity (**B**) when there were only interictal discharges. (**C**,**D**) Recordings of spiking activity and postsynaptic currents of 2 pyramidal cells in the entorhinal cortex in the control group (**C**) and under LFLS (**D**) when the first light step induced ictal discharge. (**E**,**F**) Recordings of the same pair of cells shown in (**C**,**D**) but with subthreshold (**E**) and threshold (**F**) light intensities. The holding potential in the voltage clamp was −27 mV. (**G**) Sigmoid approximation (Boltzmann fit) of the dependence of the probability to induce interictal discharges by photostimulation on light intensity. The black curve is the averaged approximation (*n* = 10), and grey curves are approximations for individual brain slices. The averaged sigmoid was calculated as $P\left(IID\right)=1-\frac{1}{1+e^{\left(LI-LI_{0}\right)/\Delta }}$, where $LI_{0}$ = 0.40 ± 0.02% of the maximum, and Δ = 0.007 ± 0.0008% of the maximum.

LFLS (0.2 Hz; duration of pulse: 100 ms; maximum intensity) suspended ictal activity in all the slices by provoking interictal discharges (*n* = 23 slices, Figure 3). Every flash induced a single interictal discharge (Figure 3B). However, it should be noted that, in seven brain slices, the first light flash provoked the development of a relatively short ictal-like discharge (Figure 3D), but with further stimulation we observed only regular interictal discharges. We also noticed that, at the beginning of stimulation, some cells showed mainly IPSCs, but EPSCs later predominated in all the neurons. The effect of LFLS was not sustained; after the cessation of LFLS, ictal discharges appeared in 180 ± 30 s.

Next, we tested the anti-ictal effect of the predominant activation of interneurons. Since the firing threshold differed between interneurons and pyramidal cells, we investigated the effect of LFLS in the threshold range from 0.3 to 0.6% of the maximum LI (Figure 3E–G). Stimulation with a LI of 0.3% was not enough to induce interictal discharge; in that case, the ictal discharges could not be suppressed (*n* = 10, Figure 3E). In the interval from 0.4 to 0.6%, the interictal discharges could be induced or not. In some slices, it depended on the iteration; in others, interictal discharges could appear during recording (see Figure 3F). When the interictal discharges appeared, there was no ictal activity. LI values higher than 0.6% effectively induced interictal discharges in all the slices (Figure 3G).

In some slices, GABAergic events were induced by subthreshold light steps (Figure 3F). Ictal activity was not suppressed if only GABAergic and not glutamatergic discharges were induced by light flashes (*n* = 5). These data suggest that the LFLS activation of inhibitory interneurons in the entorhinal cortex was not sufficient to prevent the generation of ictal discharges.

### 2.3. LFLS Activation of Parvalbumin-Positive Interneurons Did Not Prevent the Generation of Ictal Discharges

In the next series of experiments, we tested whether LFLS of one of the most common types of cortical interneurons, parvalbumin-containing (PV) fast-spiking interneurons [23,24,25], could prevent ictal activity.

**Figure 4:**
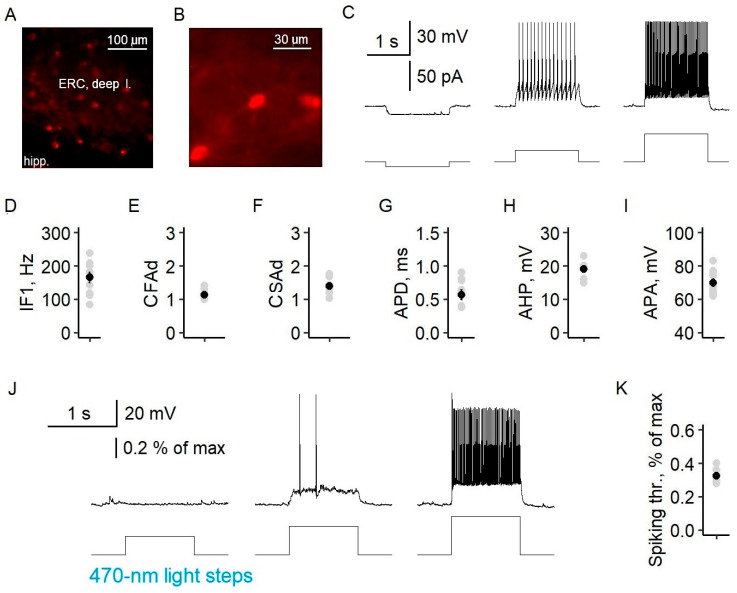
Specific expression of channelrhodopsin2 in PV interneurons of the entorhinal cortex of Model 2. (**A**) Fluorescent microphotography of the deep layers of the entorhinal cortex (ERC) of Model 2 obtained with 555 nm of light showing the efficacy of ChR2-mCherry expression (hipp.—hippocampus). (**B**) Fluorescent microphotography of a representative ChR2-mCherry neuron showing its multipolar morphology. (**C**) A representative example of mCherry neuron responses to current steps in ACSF. (**D**–**I**) Electrophysiological characteristics of PV-ChR2-mCherry neurons corresponding to fast-spiking interneurons. The plots show the 1st instantaneous frequency (IF1, (**D**)), the coefficients of fast (CFAd (**E**)) and slow adaptation (CSAd (**F**)), the action potential duration (APD (**G**)), afterhyperpolarization (AHP (**H**)), and the action potential amplitude (APA (**I**)). (**J**) Representative example of mCherry neuron responses to light steps in 4-AP epileptogenic solution. (**K**) Statistical data for the minimum light intensity inducing spiking (spiking thr.). Circles are individual values, small black circles are mean values, and whiskers are standard errors of the means.

To assess the specificity of virus expression in parvalbumin-containing fast-spiking interneurons, we examined the distribution of neurons with mCherry reporter expressions across the cell layers, as well as their electrophysiological properties. mCherry neurons were multipolar and were located in all but the first layer of the entorhinal cortex (Figure 4), which is consistent with the immunohistochemical data for PV cells [26]. Their electrophysiological characteristics corresponded well to those of fast-spiking interneurons. The neurons exhibited a high-frequency non-accommodating firing pattern with fast spikes. The instantaneous frequency was 166 ± 16 Hz (*n* = 10), and the coefficients of fast and slow adaptation were 1.14 ± 0.04 (*n* = 10) and 1.40 ± 0.07 (*n* = 9), respectively. The increase in the interspike interval during 1 s of recording did not exceed 15%. Fluorescent neurons also had an action potential duration of 0.56 ± 0.07 ms (*n* = 9), afterhyperpolarization of 19 ± 1 mV (*n* = 7), and an action potential amplitude of 70 ± 2 mV (*n* = 10). A 470 nm light pulse with a minimal intensity of 0.33 ± 0.02% of the maximum (*n* = 7) induced the action potential generation of fluorescent parvalbumin interneurons (Figure 4K).

**Figure 5:**
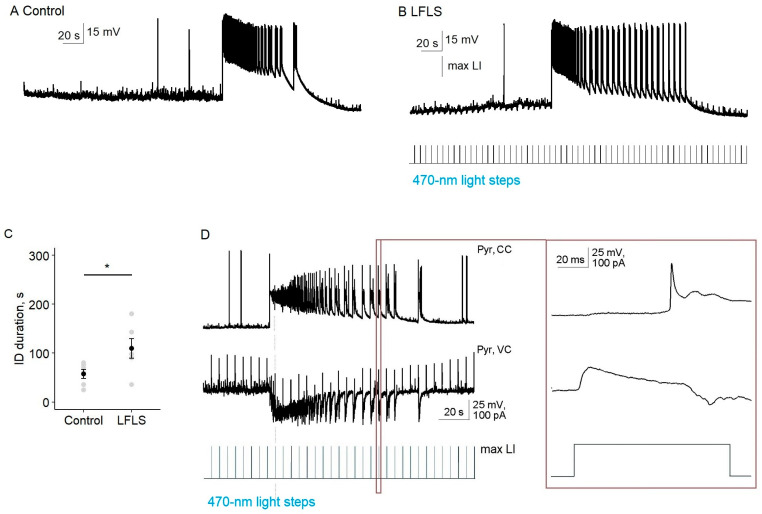
LFLS of parvalbumin interneurons in the entorhinal cortex of Model 2 did not stop ictal activity and prolonged its clonic phase. (**A**,**B**) Ictal discharges shown with spiking activity of a representative pyramidal cell in the control group (**A**) and during LFLS (**B**). (**C**) Statistical data for the duration of spiking activity during ictal discharge (ID duration, * *p* < 0.05, paired *t*-test, *n* = 6). (**D**) Representative simultaneous recording of spiking activity of one pyramidal cell and postsynaptic currents in another at the holding potential of −27 mV during LFLS. One scaled response to a light flash is shown in the right panel.

Next, we tested the effect of the LFLS activation of inhibitory neurons on ictal activity in the entorhinal cortex. We found that, in contrast to the nonspecific stimulation of neurons in Model 1, LFLS of parvalbumin-positive interneurons was insufficient to prevent the generation of ictal discharges (*n* = 9, Figure 5). Moreover, LFLS prolonged the duration of the clonic phase of the ictal discharge, so its total duration increased by 89% (56 vs. 106 s, *n* = 6, *p* < 0.05, paired *t*-test).

In the silent state, every light flash induced the hyper- or depolarization of a pyramidal cell due to the activation of PV interneurons, but it was a subthreshold for pyramidal neurons (*n* = 8). Therefore, at this stage, the activation of PV interneurons was not enough to induce interictal discharge.

In the tail of the ictal discharge, each burst of synchronized neural activity was triggered by a flash of light. We recorded synchronized GABA–glutamate currents of the postsynaptic neuron in response to each flash of light (*n* = 4, Figure 5D). Thus, the effect of LFLS of PV interneurons in the entorhinal cortex could be considered pro-epileptic in the 4-AP model.

### 2.4. Low-Frequency Hyperpolarization of CamkII-Positive Neurons Did Not Affect Ictal Activity

**Figure 6:**
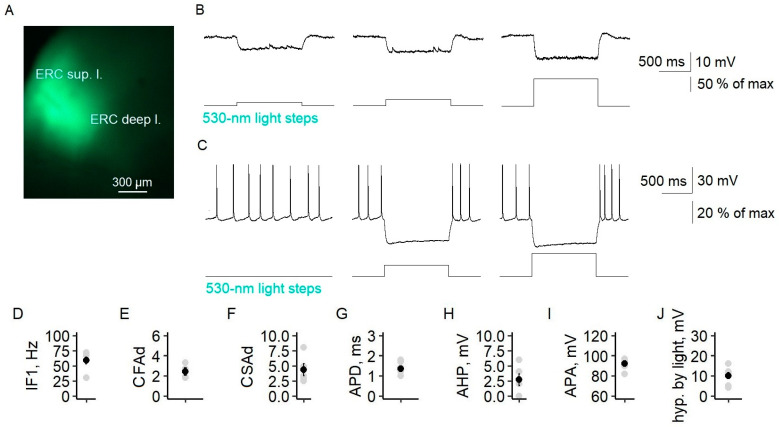
Specific expression of archaerhodopsin in CaMKII neurons. (**A**) Fluorescence micrograph of the entorhinal cortex (ERC) showing the efficiency of Arch-YFP expression. (**B**) A representative example of YFP neuron responses to light steps with different intensities. (**C**) Responses to light steps of a depolarized neuron. (**D**–**I**) The electrophysiological characteristics of YFP-positive neurons corresponded to regularly spiking pyramidal neurons: 1st instantaneous frequency (IF1 (**D**)), coefficients of fast (CFAd (**E**)) and slow adaptation (CSAd (**F**)), action potential duration (APD (**G**)), afterhyperpolarization (AHP (**H**)), and action potential amplitude (APA (**I**)). (**J**) Statistical data for the maximum hyperpolarization (hyp.) induced by light.

**Figure 7:**
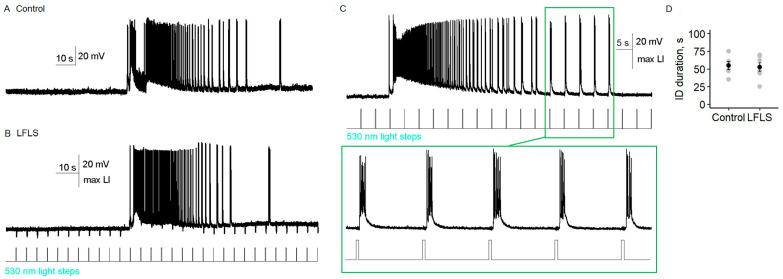
Low-frequency hyperpolarization of CaMKII neurons did not affect ictal activity in the entorhinal cortex. (**A**,**B**) Spiking activity of a representative CamKII neuron before ((**A**) control) and during LFLS (**B**). (**C**) An example of ictal discharge during photostimulation when bursts in the clonic phase happened in response to the light turning off. (**D**) Statistical data for the duration of spiking activity during ictal discharge.

In our last series of experiments, we tested whether the low-frequency hyperpolarization of principal cortical neurons could prevent the generation of ictal activity (Model 3 and Figure 6 and Figure 7). Since in epilepsy the balance of excitation and inhibition shifts towards excitation, hyperpolarization of excitatory neurons and the corresponding suppression of their activity seems to be a promising method of treatment [27]. It is known that inhibitory interneurons under certain conditions, including epileptic status, can cause the depolarization of postsynaptic neurons due to a reversal potential shift for chloride ions [28,29]. Therefore, the hyperpolarization of principal neurons through light activation of archaerhodopsin may have advantages over the activation of inhibitory interneurons.

We expressed archaerhodopsin under the promoter CaMKII in the entorhinal cortices of Wistar rats. The YFP-positive neurons had apical dendrites and triangular cell bodies. These neurons had the electrophysiological characteristics of a regular-spiking neuron, such as a first instantaneous frequency of 59 ± 6 Hz (*n* = 6) and coefficients of fast and slow adaptation of 2.4 ± 0.4 and 4.3 ± 1.0, respectively. The action potential duration was 1.4 ± 0.1 ms (*n* = 6), and the amplitude was 92 ± 2 mV (*n* = 6). In the entorhinal cortex, a flash of 530 nm of light hyperpolarized YFP-positive principal neurons, and neuron firing ceased if they were depolarized (Figure 6B,C). The maximum hyperpolarization was 10.1 ± 1.6 mV (*n* = 7) and was observed at 48 ± 7% of the maximum light intensity.

Comparing the ictal activity before and during LFLS of CaMKII neurons (*n* = 11), we did not find any significant differences (Figure 7). The duration of ictal discharge did not depend on the low-frequency activation of archaerhodopsin (*t*-test, *p* = 0.8, *n* = 7, Figure 7D). In one of the slices, bursts of spiking activity during the clonic phase of the ictal discharge occurred almost immediately after the end of the light flash (Figure 7C); however, no distinct synchronization of light flashes and activity bursts was detected in other slices (Figure 7B).

Our results suggest that the low-frequency hyperpolarization of CaMKII neurons did not have an antiepileptic effect.

Thus, of the three cases of neuron-type-specific low-frequency stimulation considered, only simultaneous activation of the excitatory and inhibitory neurons (Model 1) had an antiepileptic effect.

### 2.5. Simulation of Effect of Stimulation in Biophysically Detailed Mathematical Model of Epileptiform Activity

**Figure 8:**
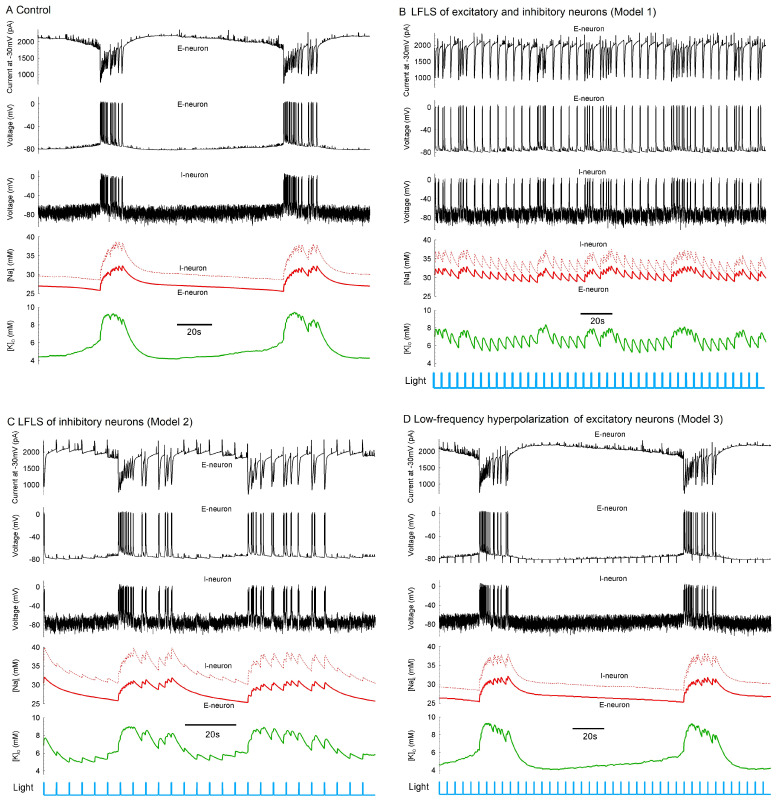
Effect of the LFLS of neurons in the mathematical model of epileptiform activity. (**A**) Spontaneously repeating ictal discharges in the control case. (**B**–**D**) To simulate the effects of LFLS as seen in experimental Models 1–3, the following current step pulses were injected: (**B**) depolarizing pulses of 25 pA in both excitatory (E) and inhibitory (I) neurons for Model 1; (**C**) depolarizing pulses of 100 pA in I neurons for Model 2; and (**D**) hyperpolarizing pulses of 25 pA in E neurons for Model 3. The signals from top to bottom are as follows: the current measured in a single E neuron in a voltage clamp at −30 mV; the membrane potentials of E and I neurons; the intracellular concentration of sodium ions; and the extracellular concentration of potassium ions.

Next, we aimed to clarify the mechanism of the observed effect of LFLS with the help of a mathematical model. In our recent publication [30], we proposed a biophysically detailed mathematical model that described mechanisms underlying the generation of ictal and interictal discharges. The model was based on the conductance-based refractory density (CBRD) approach [31,32] to describe populations of neurons, where a population was defined as a large number of similar neurons each receiving a common input and individual noise. The cortical network was modeled as three neuronal populations interacting under the conditions of dynamic changes in ionic concentrations, as previously suggested [33,34]. Two types of excitatory and one type of inhibitory cells were considered, where one excitatory population maintained normal and the other displayed an elevated level of intracellular chloride concentration, thus revealing the depolarization effect of GABA. The model reproduced repeating ictal discharges, as shown in Figure 8A. The model predicted that ictal discharge generation was determined mostly by the oscillations of the concentrations of extracellular potassium and intracellular sodium ions. A crucial role in the termination of each ictal discharge belonged to Na-K pumping. The pumping become more active at the high intracellular concentration of the sodium ions [Na^+^]_i_ that was reached during the tonic phase of ictal discharge.

Activation resulted in both an electrogenic effect of the pumping and a decrease in the extracellular concentration of potassium ions [*K*^+^]_o_, i.e., termination of the positive feedback that maintained the hyperactivated state of neuronal activity during ictal discharge. The pumping remained active after ictal discharge, thus returning [*K*^+^]_o_ to the initial level before ictal discharge, or even lower. This mechanism suggests that any event that affected [Na^+^]_i_ could affect the generation of ictal discharges through the effect on pumping. We suggest that a stimulation that provoked interictal-like events was able to elevate the mean level of [Na^+^]_i_ and, thus, affect the regime of activity. Therefore, we considered three versions of stimulation according to experimental Models 1–3.

In the case corresponding to experimental Model 1, all the neurons received 200 ms pulses of depolarizing 25 pA current at a 0.2 Hz frequency. Instead of ictal discharge generation, we observed a regime with interictal-like discharges provoked by the pulses (Figure 8B). These interictal discharges were accompanied by increments of [Na^+^]_i_ of 2 mM or so, which, in turn, increased the activation of Na-K pumping. A series of interictal discharges increased [Na^+^]_i_ such that the pumped current prevented the generation of ictal discharge. Therefore, the provocation of interictal discharges with stimulation prevented the generation of ictal discharges, which was consistent with our experiments on optogenetic stimulation.

In contrast, the stimulation of interneurons with a depolarizing current (Figure 8C) or the hyperpolarization of excitatory neurons (Figure 8D) did not provide pure interictal activity. In the case of the specific excitation of interneurons, only part of the interneurons received a depolarizing current, as shown in Figure 8C, where the interneuron was “non-fluorescent”.

The model predicted that the elevated level of [Na^+^]_i_ prevented ictal discharges and was responsible for the transfer to interictal activity. However, in our experiments, we had no possibility of measuring [Na^+^]_i_. Instead, we could measure [*K*^+^]_o_. According to the simulations, in the interictal regime, this concentration should also be maintained at an elevated level because the evoked interictal-like discharges contributed to the increase in [*K*^+^]_o_, as well as because the activation of Na-K pumping in this regime was not as strong as during and after each of the ictal discharges.

### 2.6. Extracellular Potassium Ion Concentration Increased under LFLS of Neurons

**Figure 9:**
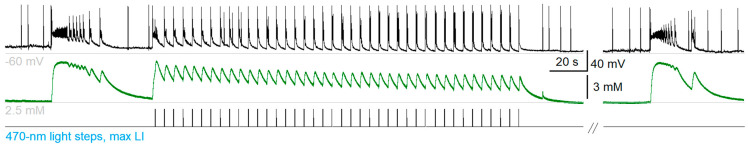
Dynamics of the extracellular potassium ion concentration before, during, and after LFLS recorded simultaneously with the spiking activity of a principal cell in the entorhinal cortex in the in vitro 4-AP model.

To test the prediction of the mathematical modeling, we recorded the extracellular potassium concentrations [*K*^+^]_o_ in the entorhinal cortices of Thy1-ChR2-YFP mice (Model 1) before, during, and after the LFLS of neurons simultaneously with the spiking activity of a principal cell located within 100 µm from a potassium-sensitive electrode (*n* = 14, Figure 9). The extracellular potassium concentration increased by 4.8 ± 0.5 mM (*n* = 14) when an ictal discharge occurred due to the spiking activity of neurons. Then, the potassium concentration decreased due to the activity of Na-K pumping, glial buffering [35], and the diffusion of potassium ions in a bath solution.

When the LFLS was switched on, the first flash could induce either interictal or ictal discharge. If the light flash induced ictal discharge, it was followed by a high jump in potassium concentration (5.0 ± 1.0 mM, *n* = 4). Otherwise, the first flash induced interictal discharges, resulting in a small increase in potassium concentration (1.9 ± 0.5 mM, *n* = 7). Each subsequent light flash induced increase in the potassium concentration by 1.5 ± 0.4 mM (*n* = 7). The extracellular potassium concentration between induced interictal discharges during the LFLS of neurons was 0.7 ± 0.5 mM (*n* = 7) higher than that between ictal discharges in the control group. When the stimulation was switched off, the extracellular potassium concentration recovered after 27 ± 4 s (*n* = 7).

Therefore, [*K*^+^]_o_ was maintained at an elevated level during the LFLS of excitatory and inhibitory neurons in experimental Model 1, as the mathematical modeling predicted. The model showed that [*K*^+^]_o_ continued to be enhanced when the pumping current was strong (Figure 9A). This result supports the hypothesis of a Na-K-pumping-dependent mechanism of LFLS action on Thy1-positive neurons.

## 3. Discussion

In search of an effective and safe treatment for pharmacoresistant temporal lobe epilepsy, in this work we studied the effects of three types of LFLS on epileptiform activity in an in vitro 4-AP model: (1) the activation of excitatory and inhibitory neurons (Model 1); (2) the activation only of inhibitory PV interneurons (Model 2); and (3) the hyperpolarization of excitatory neurons (Model 3). Only in the first model did LFLS effectively suppress ictal activity. During the LFLS of excitatory and inhibitory neurons, ictal activity was replaced by interictal activity. An antiepileptic effect of low-frequency stimulation has already been observed in a number of studies [8,9,18].

This effect has been observed with low-frequency electrical stimulation for in vitro models of temporal lobe epilepsy [8,9]. Barbarosie and Avoli showed for the first time that ictal activity in entorhinal cortex–hippocampal slices of adult mice induced by a magnesium-free solution turned into interictal activity during low-frequency electrical stimulation of the CA1 region of the hippocampus. A similar shift from ictal activity to interictal activity in the perirhinal cortex was observed in a 4-AP model of rat brain slices during electrical stimulation of the lateral nucleus of the amygdala [9].

Using an optogenetic approach, Shiri et al. showed that the selective LFLS of CaMKII neurons in the entorhinal cortices of juvenile mice in an in vitro 4-AP model of temporal lobe epilepsy, as well as the LFLS of PV or somatostatin interneurons, had an antiepileptic effect, which consisted of a reduction in ictal discharge frequency and duration [18]. In our study, using Model 1, we were able to completely suppress ictal activity, but the activation of only PV interneurons in the second model proved to be ineffective. Moreover, we assumed that the activation of only principal neurons would be sufficient to suppress ictal activity, but so far, we have only model calculations in favor of this (Appendix C).

The discrepancy between the results of our experiment on PV-Cre mice and those of Shiri et al. may be due to a difference in experimental design. Since Shiri et al. injected the viral construct on postnatal day 15, they could obtain almost ubiquitous ChR2 expressions in PV interneurons in the entorhinal and perirhinal cortices. We performed the study on adult mice (aged 3–12 months), so the ChR2 expression in PV interneurons was more local. Shiri et al. performed their experiments on young adult animals (aged 30–40 days). We previously revealed that, in a 4-AP model, ictal activity in juvenile and adult rats proceeded differently [36], and low-frequency electrical stimulation in brain slices of juvenile animals leads to a decrease in the frequency of ictal discharges, while in adult rats ictal activity is replaced by provoked interictal activity [20]. Another methodical difference is the different LFLS protocols. Shiri et al. used a stimulation protocol lasting 180 s with a frequency of 1 Hz, whereas we used a longer LFLS duration (350 s) with a lower frequency (0.2 Hz).

By reducing the intensity of the LFLS in the first model, we found that elicited interictal activity was a mandatory condition for the antiepileptic effect. In the case when the light intensity was insufficient to induce interictal discharge, ictal discharges were generated. In order to comprehensively describe the mechanisms of ictal and interictal discharge generation and LFLS action, we used the mathematical model proposed in our previous works [33,34,37] and supplemented it with a module reflecting LFLS action. The proposed model belongs to the class of biophysically detailed models in which the excitation of the neuronal network is described in the form of equations for the conductance of voltage-dependent and ligand-dependent ion channels, where the excitation–inhibition balance changes over time according to the dynamics of ion concentration. Our model is one of the few models of this class that can reproduce spontaneously repeated ictal and interictal discharges. For example, Krishnan et al. [38] and Gentiletti et al. [39] reproduced the ion dynamics and neuronal excitation during single ictal discharges, and just as in our model, Na-K pumping was suggested to play a crucial role in discharge termination. As in our model, Wei et al. reproduced a regime of the continuous generation of ictal discharges, also with the dominant action of Na-K pumping, but mediated by the slow dynamics of oxygen concentration [40].

In general, our model showed consistent results in comparison with experiments and known models, which has been reflected in our previous publications focused on the generation of GABAergic and GABA–glutamatergic interictal discharges [34], their propagation [33], the generation of ictal discharges [30], and the slow propagation of the ictal discharge front [37]. In particular, the model reflects known data on the dynamics of ionic concentrations during ictal discharges, when the maximum [*K*^+^]_o_ is observed in the early phase of discharge development, the maximum [Na^+^]_i_ is observed in the final phase, and the [Cl^−^]_i_ qualitatively repeats the dynamics of [*K*^+^]_o_ and modulates the form and type of interictal-like discharges between and during ictal discharges. The detailed modeled mechanisms of discharge generation were presented as a minimal Na-K-pump-based model, “Epileptor-2” [21]. The consistency of the model with the experiments allowed us to formulate some hypotheses about the mechanism of action of LFLS.

Based on mathematical modeling, we assumed the following mechanism by which evoked interictal activity prevented the generation of ictal discharge: (1) evoked interictal discharge led to increased [Na^+^]_i_ due to the activation of glutamate receptors and the generation of action potentials; and (2) it activated Na-K pumping, which captured potassium ions from the extracellular space and, thus, prevented a sharp rise in [*K*^+^]_o_, which was necessary for the generation of ictal discharge.

Experimental data proving that Na-K pumping can be a target for antiepileptic action were also recently obtained using the Na-K-pump-activating antibody DRRSAb [41,42]. The activation of Na-K pumping in vivo affected seizure susceptibility in epilepsy models caused by pilocarpine [41] and pentylenetetrazol (PTZ) [42].

An important argument in defense of the proposed mechanism of the antiepileptic action of Na-K pumping is that [*K*^+^]_o_ was higher between the interictal discharges induced by LFLS than between ictal discharges in the control group. If the detected antiepileptic properties of low-frequency activation of the excitatory and inhibitory neurons by light could only be explained by the maintenance of Na-K-pumping activity, then alternative ways of maintaining its activity could be suggested as a potential treatment for epilepsy. Although light-induced interictal activity is less damaging to nerve tissue than ictal activity [43], it also has undesirable side effects. The direct activation of Na-K pumping in the epileptic foci could be an effective option. For this purpose, it is possible to use approaches with changes in the ionic concentrations in epileptic tissue [44]. Prototypes of suitable pharmacological agents used to modulate Na-K-pumping activity could be, for example, DRRSAb [42] or monensin [45].

## 4. Materials and Methods

### 4.1. Animals

All animal procedures followed the guidelines of the European Community Council Directive 86/609/EEC and were approved by the Animal Care and Use Committee of Sechenov Institute of Evolutionary Physiology and Biochemistry of the Russian Academy of Sciences. For optogenetic experiments, Thy1-ChR2-YFP (*n* = 36, aged 3–12 months, founder line 18; stock #007612, Jackson Laboratory, Bar Harbor, ME, USA) and PV-Cre (*n* = 7, aged 3–12 months, stock #017320, Jackson Laboratory,) mice lines, as well as 21-day-old Wistar rats were used. Both male and female animals were used for experiments.

### 4.2. Stereotaxic Virus Injections

AAV-EF1α-double-floxed-hChR2(H134R)-mCherry was prepared at the Institute of Bioorganic Chemistry RAS (Moscow, Russia) from plasmid pAAV-EF1a-double-floxed-hChR2(H134R)-mCherry-WPRE-HGHpA (#20297, Addgene) with a titer of 2.4 × 10^12^ GC/mL. PV-Cre mice were anesthetized with a cocktail of zoletil-100 (40 mg/kg) and xylazine (10 mg/kg) and positioned in a stereotaxic frame (SM-15 Narishige, Tokyo, Japan). AAV-EF1α-double-floxed-hChR2(H134R)-mCherry vector was delivered to the entorhinal cortex (1 µL at a rate of 0.05 µL/min). Injection coordinates were as follows: anteroposterior: –4.0; mediolateral: 3.5; and dorsoventral: 4.2 mm. Hamilton syringes were inserted at a 3–5° anteroposterior angle. After completion of the surgery, mice were placed on a heat pad for 24 h to allow recovery and then returned to their home cages. The seams were treated with laevomecolum for a week.

AAV-CamkIIa-eArch3.0-EYFP was prepared at Peter the Great St. Petersburg Polytechnic University (St. Petersburg, Russia) from pAAV-CamkIIa-eArch3.0-EYFP (Addgene, #35516) with a titer of 10^12^ GC/mL. Wistar rats of 21 days of age were anesthetized with a cocktail of zoletil-100 (20 mg/kg) and xylazine (10 mg/kg). Then, rats were placed in a stereotaxic frame (SM-15 Narishige). After removal of the skin and cleaning of the skull, a hole was drilled above the left entorhinal cortex (coordinates: anteroposterior: −8.0; mediolateral: 6.0; and dorsoventral: 6 mm). A Hamilton syringe was used to inject 1.5 µL AAV. After completion of the surgery, pups were placed on a heat pad for 24 h to allow recovery and then returned to their home cages. The seams were treated with laevomecolum for a week.

### 4.3. Histology

Horizontal 100 μm thick slices containing the entorhinal cortex and hippocampus were cut using a Microm HM 650 V vibratome (Thermo Scientific, Waltham, MA, USA) for fluorescence imaging. The distributions of YFP- or mCherry-marked neurons in slices were analyzed with a DMI6000B microscope (Leica Microsystems, Wetzlar, Germany) equipped with 488 nm and 555 nm light sources and L5 and N3 filter cubes (Leica Microsystems, Wetzlar, Germany).

### 4.4. Slice Preparation

The brains were quickly removed and immersed in ice-cold sodium-free artificial cerebrospinal fluid (ACSF) (in mM: 110 N-Methyl-D-glucamine, 2.5 KCl, 1.2 NaH_2_PO_4_, 10 MgSO_4_, 0.5 CaCl_2_, 25 NaHCO_3_, and 25 dextrose; pH was adjusted to 7.3–7.4 with HCl). The osmolarities of extracellular solutions were adjusted to 300–310 mOsm. All the used solutions were aerated with carbogen (95% O_2_, 5% CO_2_). Horizontal 300 μm thick slices were cut using a Microm HM 650 V vibratome. Slices contained the entorhinal cortex and hippocampus, but the CA3 zone was removed to observe stable ictal activity.

After, cut slices were transferred into solution with the following composition (in mM): 116 NaCl, 10 HEPES, 2.5 KCl, 1.25 NaH_2_PO_4_, 1 MgSO_4_, 2 CaCl_2_, 24 NaHCO_3_, 13 D-glucose, and 0.1 mM 4-AP (pH was adjusted to 7.3–7.4 with NaOH). Slices were incubated for 10 min at 35 °C and then for 1 h at room temperature.

### 4.5. Whole-Cell Recordings

Recordings were performed at 30 °C in epileptogenic solution with the following composition (in mM): 125 NaCl, 3.5 KCl, 1.25 NaH_2_PO_4_, 0.25 MgSO_4_, 2 CaCl_2_, 24 NaHCO_3_, 13 D-glucose, and 0.1 4-AP. The flow rate in the perfusion chamber was 5–6 mL/min. The liquid junction potentials were measured as described [46], and the holding potential was compensated offline for voltage clamp recordings by subtracting 7 mV.

Neurons were visualized using a Nikon Eclipse FN1 microscope (Nikon; Tokyo, Japan) equipped with differential interference contrast optics, a Grasshopper3 video camera (FLIR Systems; Wilsonville, OR, USA), an optical filter set for the detection of fluorescent light (YFP MXR00107 and TXRed MXR00109 filter cubes, Semrock, West Henrietta, NY, USA), and BLS-LCS-0505-14-22 and BLS-LCS-0560-03-22 High-Power LED Collimator Sources with 135 and 240 (mW)^3^ maximum output power, respectively (Mightex Systems, North York, ON, Canada).

Patch electrodes (3–5 MΩ) were pulled from borosilicate-filamented glass capillaries (World Precision Instruments, Sarasota, FL, USA) with a P-1000 Micropipette Puller (Sutter Instrument, Novato, CA, USA). A potassium-gluconate-based filling solution was used for current clamp recordings (in mM): 135 K-gluconate, 10 NaCl, 5 EGTA, 10 HEPES, 4 ATP-Mg, and 0.3 GTP. For the voltage clamp recordings, we used a CsMeS-based pipette solution (in mM): 127 CsMeS, 10 NaCl, 5 EGTA, 10 HEPES, 6 QX314, 4 ATP-Mg, and 0.3 GTP. The osmolarities of pipette solutions were adjusted to 290–300 mOsm, and pH was adjusted to 7.25.

Whole-cell recordings were performed with a HEKA EPC-10 double amplifier (HEKA, Harvard Bioscience, Inc., Holliston, MA, USA) using PatchMaster 1.2 software (HEKA). The data were filtered at 10 kHz and sampled at 33 kHz. Access resistance was less than 20 MΩ and remained stable during the experiments (≤30% increase) in all cells included in the analysis.

### 4.6. Analysis of Electrophysiological Characteristics

For the analysis of electrophysiological properties of neurons, 1.5 s current steps with different amplitudes were applied to each neuron. The firing pattern properties were estimated for the train with a half-maximal firing rate using the following set of parameters:

(1) The first instantaneous frequency (*IF*1, in Hz), as the reversed interval between the second and first spikes:

$$IF1=\frac{1}{t_{2}-t_{1}},$$

(2) The coefficient of fast-firing frequency adaptation (*CFAd*):

$$CFAd=\frac{t_{3}-t_{2}}{t_{2}-t_{1}},$$

(3) The coefficient of slow-firing frequency adaptation (*CSAd*):

$$CSAd=\frac{t_{n}-t_{n-1}}{t_{2}-t_{1}},$$

where *t_i_* is moment of overcoming the threshold of the *i*th spike, and *n* is the number of spikes in the spike train with the half-maximal firing rate. The interspike interval (*ISI_i_*) was calculated as follows:

$$ISI_{i}=t_{i+1}-t_{i}.$$

Single spike properties were estimated for the first spike in the train with the half-maximal firing rate. The action potential amplitude (APA) was measured from the threshold to the peak. The threshold was estimated as the membrane potential at the point at which the interpolated rate of voltage increase (dV/dt) reached 10 mV/ms. The action potential duration (APD) was the spike width at its half-maximal amplitude. The amplitude of afterhyperpolarization (AHP) was measured from the threshold to the greatest negative membrane potential after the action potential.

### 4.7. Recording of Extracellular Potassium Concentration

Measurement of the extracellular potassium concentration was performed with K^+^-selective microelectrodes manufactured and calibrated as described previously [37]. In brief, electrodes were pulled from borosilicate glass (Sutter Instrument). The inner wall of a micropipette was then silanized using hexamethyldisilazane vapor (Sigma Aldrich, St. Louis, MO, USA) at 225 °C for 90 min. Before the experiment, the micropipette was filled with 100 mM KCl solution and then backfilled with valinomycin (potassium ionophore I, cocktail A, Sigma Aldrich). The recording of electrode voltage was performed using a HEKA 10 USB patch-clamp amplifier in current-clamp mode. We checked the stability of the electrodes at the start and end of each recording. Data from unstable electrode recordings were discarded. The extracellular K^+^ concentration ([*K*^+^]_o_) at a given moment (*t*) was calculated from the electrode voltage (*V*(*t*)) as follows:

$${\left[K^{+}\right]}_{o}\left(t\right)=2.5\;e^{S*V\left(t\right)}$$

where *S* is the scaling factor, which was estimated by applying solutions with different [*K*^+^]_o_ at the tips of ionophore-filled electrodes using a fast application system (HSSE-2/3, ALA Scientific Instruments Inc., Farmingdale, NY, USA). In all electrodes tested, the scaling factor was within a small range (0.043–0.045), so for all obtained recordings, *S* was set as equal to the average value of 0.044 mV^−1^.

### 4.8. LFLS

For ChR2 excitation, blue light (470 nm) was delivered from an LCS-0470-03-22 BLS-Series High-Power LED Collimator Source with a 200 mW maximum output power (Mightex Systems) connected to the epi-illumination port of a microscope. The beam was deflected by a 610 nm dichroic mirror in an empty filter cube and conveyed to the slice through a 40× water immersion objective. We measured with Megeon 21010 luxmeter that the maximal illuminance (100%) at the surface was 36500 Lux.

For archaerhodopsin activation, green light (530 nm) was delivered from an LCS-0530-03-22 BLS-Series High-Power LED Collimator Source with a 290 mW maximum output power (Mightex Systems). The maximal illuminance (100%) at the surface of the slice was 1900 Lux.

The protocol for LFLS included light pulses with 100 or 200 ms durations. To determine the threshold intensity of the 470 nm photostimulation for the depolarization and spiking of ChR2 neurons, we applied 1 s steps of light with different intensities. The same protocol was used to determine the level of hyperpolarization of neurons through the 530 nm stimulation of CamkII-Arch-YFP neurons.

### 4.9. Statistical Analysis

Statistical analyses was performed using RStudio 1.0.136 (RStudio Inc., Boston, MA, USA) software. The normality of the sample data was evaluated with the Shapiro–Wilk test. Student’s *t*-test or one-way ANOVA with Tukey’s post hoc tests were used for normally distributed data. The results are expressed as mean ± standard error of the mean, where *n* is the number of brain slices in the group.

### 4.10. Simulations

The mathematical model was based on the CBRD approach [31,32]. For a single neuronal population, the approach allowed the calculation of the instantaneous firing rate using known synaptic conductance and reversal potential values as the input signals. The applied model of epileptiform activity consisted of three populations and ionic dynamic equations, as described in [34]. Spontaneous background activity of inhibitory neurons needed for chloride accumulation inside neurons was evoked by extra noise that implicitly mimicked the spontaneous synaptic activity observed in the experiment after 4-aminopyridine application.

## Figures and Tables

**Figure A2:**
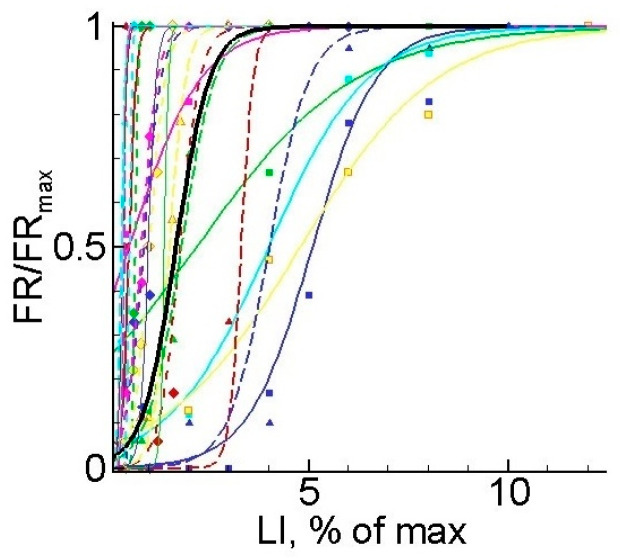
The dependence of the normalized firing rate (*FR*) on light intensity for individual pyramidal neurons (colored points reflect experimental data, colored curves are Boltzmann fits for each cell (*n* = 15), and black curve is averaged Boltzmann fit. The averaged Boltzmann fit was calculated as $FR=FR_{max}\left(1-\frac{1}{1+e^{\left(LI-1.68\right)/0.42}}\right)$.

**Figure A3:**
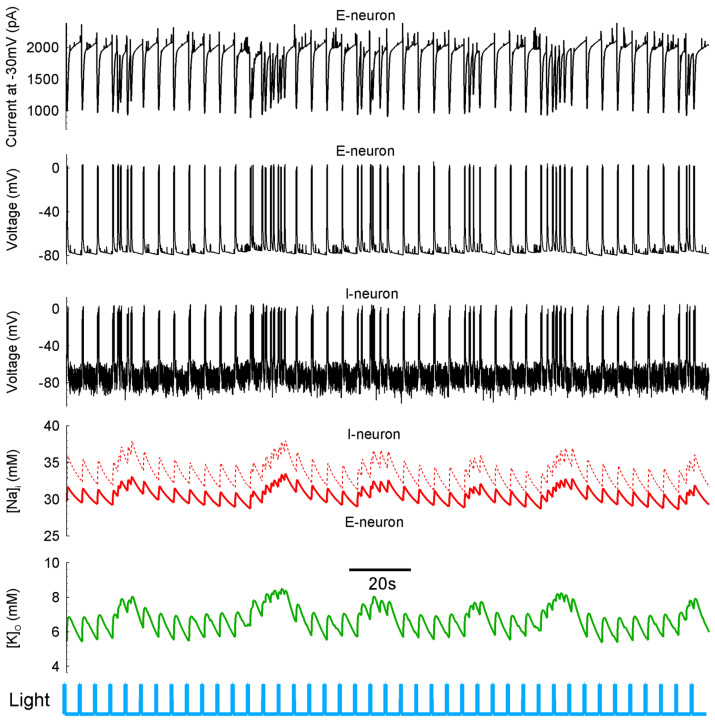
Effect of the LFLS of pyramidal neurons in the mathematical model of epileptiform activity. The control case is presented in Figure 8A. To simulate the effect of LFLS on pyramidal cells, current step pulses with an amplitude 25 pA were injected in excitatory neurons.

## Data Availability

The data presented in this study are available on request from the corresponding author.

## Appendix B

An increase in light intensity raised the frequency of neuron action potentials. The FR–LI curve was well-fitted with the Boltzmann sigmoid function. We found that the light intensity for the half-maximal firing rate was 1.68% of the maximum.
